# Inactivation of *Listeria monocytogenes* and *Salmonella* Typhimurium by piezoelectric cold plasma: oxidative, structural and metabolic pathways

**DOI:** 10.1007/s00203-025-04589-5

**Published:** 2025-12-08

**Authors:** Y. K. Oliinychenko, B. K. Tiwari, A. Ch. Stratakos

**Affiliations:** 1 Centre for Research in Sustainable Agri-food & Environment, School of Applied Sciences, University of the West of England, Coldharbour Ln, Bristol, BS16 1QY UK; 2 Department of Food Biosciences, Teagasc Food Research Centre, Teagasc, Ashtown, Dublin 15, D15 DY05 Ireland

**Keywords:** Nonthermal processing, Foodborne pathogens, Pathogen inactivation, Cold atmospheric plasma

## Abstract

Cold atmospheric plasma (CAP) is an innovative, non-thermal decontamination technology with promising applications in food safety. This study investigated the antimicrobial mechanisms of CAP against the foodborne pathogens *Salmonella* Typhimurium and *Listeria monocytogenes*. CAP was applied using piezoelectric direct discharge (PDD) technology for 0, 1, 6, 9, and 15 min to simulate nonthermal decontamination conditions relevant to food processing. Antimicrobial effectiveness was evaluated using culture-based enumeration, while CAP-induced cellular damage was assessed using scanning electron microscopy (SEM), and assays measuring lipid peroxidation, reactive oxygen species accumulation, membrane permeability and malate dehydrogenase activity. Bacterial viability significantly decreased by up to 5.7 log CFU/mL after 6 min and ≤ 6.6 log CFU/mL after 9 and 15 min treatments, compared with untreated pathogens. These reductions were accompanied by corresponding increases in membrane permeability of 50%, 65%, and 94%, respectively. Culture-based enumeration confirmed reductions of ~ 4.5 log CFU/mL after 6 min and ≤ 6.5 log CFU/mL following 9 and 15 min treatments. CAP treatment for at least 6 min significantly elevated intracellular reactive oxygen species, which triggered lipid peroxidation as evidenced by elevated malondialdehyde and peroxide values. Furthermore, CAP treatment resulted in a significant reduction in malate dehydrogenase activity, indicating disruption of cellular metabolic function. SEM supported CAP-induced cellular alterations by revealing morphological damage, including porosityshrinkage and cytoplasmic leakage. Overall, PDD-generated CAP induced a multi-targeted antimicrobial effect, supporting its potential as a nonthermal decontamination method in food processing. Further research should focus on its application to a range of food matrices and its impact on quality parameters.

## Introduction

Foodborne illnesses remain a significant global health concern, causing an estimated 600 million cases of illness and 420,000 deaths each year, along with substantial economic losses worldwide (Lee and Yoon 2021; Scharff 2015). In the United States alone, these losses are estimated at USD 55.5 billion per year, primarily due to healthcare expenses (Scharff 2015). Among the major pathogens responsible, *Salmonella* spp. and *Listeria monocytogenes* remain particularly prevalent, underscoring their significance in food safety management (Lianou et al. 2017). Although conventional preservation methods remain effective, the rising global population and intensification of food production have contributed to an increase in foodborne outbreaks, thereby highlighting the need for supplementary processing interventions (Sarno et al. 2021; van Dijk et al. 2021). In addition, the growing consumer preference for minimally processed, ‘clean label’ products with fewer chemical additives has increased demand for alternative, nonchemical decontamination methods (Hüppe and Zander 2021). Collectively, these challenges underscore the importance of developing innovative technologies capable of ensuring microbial safety, maintaining product quality, achieving sustainability, and aligning with evolving consumer expectations.

Cold atmospheric plasma (CAP) is a novel nonthermal technology being studied for microbial inactivation in food processing (Jayasena et al. 2023). CAP offers several advantages over conventional thermal and chemical decontamination methods, including low-temperature operation, absence of chemical residues, minimal impact on food quality traits, energy efficiency and environmental sustainability (Jayasena et al. 2023; Scharff 2015). However, the broader industrial application of CAP remains limited, mainly due to an incomplete mechanistic understanding of its antimicrobial effectiveness across diverse microbial species, food matrices and treatment conditions. A comprehensive analysis of CAP–bacteria and CAP–matrix interactions is essential to develop standardised treatment protocols, optimise antimicrobial performance and facilitate industry integration.

Dielectric barrier discharge (DBD) and plasma jet technology are the most frequently studied systems for CAP generation for food-related applications (Jayasena et al. 2023). DBD technology generates plasma through the application of high voltage across electrodes that are separated by a dielectric barrier, thereby stabilising the discharge and inhibiting arcing (Chirokov et al. 2005). Plasma jet technology produces a continuous plasma discharge by channeling gas flow between powered and grounded electrodes (Reema, Khanikar et al. 2022). These CAP treatment technologies significantly increase levels of reactive oxygen species (ROS), malondialdehyde (MDA), and hydrogen peroxide in foodborne pathogens such as *Escherichia coli*, *Salmonella enteritidis*, and *Listeria monocytogenes* (Hu et al. 2025; Lavrikova et al. 2025; Patange et al. 2019). In addition to inducing oxidative damage, CAP altered bacterial metabolism by reducing malate dehydrogenase (MDH) activity in a time-dependent manner following DBD treatment in *Salmonella enteritidis* and *Listeria monocytogenes* (Fu et al. 2022). Furthermore, increased membrane permeability and nucleic acid leakage observed in *Listeria monocytogenes* and *Salmonella* Typhimurium indicate compromised membrane integrity (Pan et al. 2023; Yadav and Roopesh 2022).

Recently, piezoelectric direct discharge (PDD) has emerged as an effective method for generating CAP in food processing applications (González-González et al. 2021; Oliinychenko et al. 2024, 2025). The piezoelectric transformer in PDD converts low-voltage alternating current into mechanical oscillations, subsequently transforming these into high-voltage plasma (Timmermann et al. 2021). The compact design, energy efficiency, and proven antimicrobial effectiveness on food contact surfaces underscore its applicability for industrial food processing (González-González et al. 2021; Oliinychenko et al. 2024, 2025; Timmermann et al. 2021). Relative to conventional DBD and plasma jet systems, the PDD source uses a piezoelectric transformer to derive high voltage from a low-power supply, without the need for additional gas supply and high-voltage hardware, which yields a compact, energy-efficient handheld application (Korzec et al. 2020a; Timmermann et al. 2021). These features simplify on-site treatment of irregular food-contact surfaces while delivering antimicrobial effects comparable to DBD/jet reports on common foodborne pathogens, offering practical advantages in portability, infrastructure and operator safety for food processing environments (González-González et al. 2021; Oliinychenko et al. 2024, 2025).

However, despite their proven effectiveness against foodborne pathogens, the underlying mechanisms of PDD-generated CAP remain limitedly studied (González-González et al. 2021; Oliinychenko et al., 2024). Given the practical advantages of PDD, further multi-parametric studies are crucial to exploring its effects on bacterial physiology and structure, particularly in the context of food safety applications.

Moreover, bacterial susceptibility to CAP has previously been attributed to differences in the cell wall morphology between Gram-positive and Gram-negative species (Das et al. 2022; Mai-Prochnow et al. 2016). Specifically, susceptibility to CAP treatment has been linked with peptidoglycan thickness, with the Gram-positive *Bacillus subtilis* demonstrating greater susceptibility than the Gram-negative *Pseudomonas libanensis* with a thinner cell wall (Mai-Prochnow et al. 2016). However, accumulating evidence indicates that CAP effectiveness extends beyond structural characteristics of the cell wall, also being influenced by treatment conditions and bacterial growth state (Kim and Min 2018), leading to species-specific modes of action (Han et al. 2016). For example, following DBD-generated CAP treatment, *Escherichia coli* undergoes membrane disruption due to lipopolysaccharide degradation, whereas *Staphylococcus aureus* exhibits intracellular damage while retaining an intact cell envelope (Han et al. 2016). The observed differences reflect the complexity of CAP inactivation and expose a knowledge gap, as mechanistic understanding has largely been shaped by DBD studies, whereas PDD-based studies remain insufficiently studied.

This study aimed to investigate the cellular mechanisms underlying bacterial inactivation by cold atmospheric plasma generated using piezoelectric direct discharge technology. To achieve this, a combination of microbiological, biochemical, and imaging methods was employed to assess changes in viable cell counts, membrane integrity, oxidative stress induction, metabolic activity, and cellular morphology in *Salmonella* Typhimurium and *Listeria monocytogenes* following CAP treatment.

## Materials and methods

### Sample preparation and treatment protocols

#### Bacterial culture preparation

Frozen stock cultures of *Salmonella* Typhimurium (NCTC 112.19) and *Listeria monocytogenes* (WDCM 00021) were streaked onto Tryptone Soya Agar (TSA; Oxoid, UK) and incubated at 37 ° C for 24 h to obtain isolated colonies. A single colony from each plate was aseptically transferred into 10 mL of Brain Heart Infusion (BHI) broth (Oxoid, UK) and incubated at 37 ° C for 24 h under aerobic conditions.

Following incubation, bacterial suspensions were centrifuged at 6500 × *g* for 10 min to pellet the cells. The supernatant was discarded, and the resulting cell pellets were washed and resuspended in 10 mL of sterile 0.8% (w/v) peptone water (Oxoid, UK) for subsequent experimental use.

#### Cold atmospheric plasma treatment

CAP treatment was applied using a piezoelectric direct discharge generator (PiezoBrush^®^ PZ3; Relyon Plasma GmbH, Germany) operated at an input voltage of 24 V AC-DC adapter that drives a CeraPlas^®^F piezoelectric transformer at ~ 50 kHz to generate atmospheric air plasma; the internal fan supplies 8 L/min airflow (no additional gas supply). Korzec et al. (2020a, b) provides a detailed schematic representation of the generator (Korzec et al. 2020a, b). Treatment durations ranged from 0 to 15 min and are specified in the relevant experimental subsections.

### Evaluation of bacterial viability

#### Enumeration of viable cells using culture-based methods

Viable counts of *S.* Typhimurium and *L. monocytogenes* were determined using selective agar plating. *S.* Typhimurium was enumerated on Xylose Lysine Deoxycholate (XLD) agar (Oxoid, UK) following incubation at 37 ° C for 24 h, while *L. monocytogenes* was quantified on ISO-standard Chromogenic Listeria Agar (Oxoid, UK) after incubation at 32 ° C for 48 h. Bacterial concentrations were expressed as logarithmic colony-forming units per mL (log CFU/mL). The detection limit of this method was 1 log CFU/mL.

### Oxidative stress and lipid peroxidation assays

#### Reactive oxygen species quantification

Intracellular ROS levels in *S.* Typhimurium and *L. monocytogenes* were quantified using 6-carboxy-2′,7′-dichlorodihydrofluorescein diacetate (H₂DCFDA; Merck, UK), following the method described by Yadav and Roopesh (2022). A 150 µL aliquot of CAP-treated or untreated bacterial suspension was mixed with 50 µL of H₂DCFDA solution to yield a final dye concentration of 20 µM. The mixture was incubated in the dark at 37 ° C for 15 min. The assay was performed in a 96-well flat-bottom cell culture microplate (Corning™ Costar™, USA), and fluorescence was measured using a microplate reader (VANTAstar; BMG LABTECH, Germany) with excitation and emission wavelengths set at 485 nm and 525 nm, respectively.

#### Lipid peroxidation using malondialdehyde assay

Lipid peroxidation in bacterial cells, indicated by MDA production, was quantified using the Lipid Peroxidation Assay Kit (MAK085; Sigma-Aldrich, UK) in accordance with the manufacturer’s protocol. Briefly, a lysis buffer containing Butylated Hydroxytoluene (BHT) was added to CAP-treated or untreated bacterial suspensions to prevent artifactual oxidation. The mixture was centrifuged at 10,000 × *g* for 10 min, and the resulting supernatant was collected.

The supernatant was then reacted with Thiobarbituric Acid (TBA) reagent and incubated at 95 ° C for 60 min to facilitate formation of the malondialdehyde–thiobarbituric acid (MDA–TBA) adduct. Fluorescence was subsequently measured using a microplate reader (VANTAstar; BMG LABTECH, Germany) at excitation and emission wavelengths of 532 nm and 553 nm, respectively. MDA concentrations (µM) were determined from a standard calibration curve prepared using known MDA concentrations.

#### Peroxide value determination

Peroxide levels (PV) in bacterial suspensions were quantified using the Peroxide Assay Kit (MAK311; Sigma-Aldrich, UK), following the manufacturer’s protocol. CAP-treated or untreated bacterial samples were mixed with the detection reagent and incubated for 30 min at room temperature. Absorbance was measured at 540–610 nm using a microplate reader (VANTAstar; BMG LABTECH, Germany). Peroxide values were calculated based on the absorbance readings, using the kit’s standard calibration curve.

### Assessment of bacterial metabolic activity, membrane integrity, and morphology after CAP treatment

#### Evaluation of metabolic activity using MDH assay

MDH activity in bacterial suspensions was assessed using the Malate Dehydrogenase Assay Kit (Sigma-Aldrich, USA), following the manufacturer’s protocol. Bacterial inoculum samples were added to the reaction mixture containing the MDH enzyme mix. Absorbance at 450 nm was recorded at 37 ° C at time zero (T₀) and subsequently at 5 min intervals for up to 30 min. The change in absorbance (ΔA₄₅₀) was corrected by subtracting the blank and converted to Nicotinamide Adenine Dinucleotide (NADH) concentration (nmol) using a standard calibration curve. MDH activity was calculated according to Eq. (1):

1 $$MDH~Activity = \frac{{S_{a} }}{{\mathrm{Re} action~time~x~S_{v} }}$$

Sa = amount of NADH (nmol) generated in the unknown sample well between T initial and T final from the standard curve.

Reaction time = T final – T initial (min).

Sv = sample volume (mL) added to well.

#### Membrane integrity assessment

Bacterial membrane integrity was evaluated using propidium iodide (PI; Merck, UK), following the protocols described by Yadav and Roopesh (2022) and Kirchhoff and Cypionka (2017). A 25 µL aliquot of PI solution was added to 150 µL of CAP-treated or untreated bacterial suspension to achieve a final dye concentration of 20 µM. The mixture was incubated in the dark at room temperature for 15 min. Fluorescence was recorded using a microplate reader (VANTAstar; BMG LABTECH, Germany) with excitation and emission wavelengths set at 488 nm and 645 nm, respectively.

#### Morphological characterization

Bacterial surface morphology was examined using a field emission scanning electron microscope (FEI Quanta 650; Thermo Fisher Scientific, USA), following the protocol described by Qian et al. (2020). Bacterial suspensions were initially fixed in 3% glutaraldehyde (Sigma-Aldrich, USA) for 1 h at room temperature to preserve cellular morphology. Samples were subsequently centrifuged and washed three times with phosphate-buffered saline (PBS; Sigma-Aldrich, USA) to remove excess fixative.

A secondary fixation was performed using 1% Osmium Tetroxide (Sigma-Aldrich, USA), followed by sequential washing and dehydration through a graded ethanol (30% to 100%) series. Complete drying was achieved by treating the samples with Hexamethyldisilazane (HMDS; Sigma-Aldrich, USA). The dried specimens were mounted on aluminium stubs, sputter-coated with a thin layer of gold, and visualised under low vacuum at 80,000× magnification using an acceleration voltage of 1–2 kV. Since the preparation artefacts can arise in the samples as a result of uneven washing, non-uniform vortexing, or the choice and concentration of fixative, images were treated as qualitative support, and all handling steps were standardised to minimise misinterpretation.

### Statistical analysis

Statistical significance between experimental groups was evaluated using one-way analysis of variance (ANOVA). Post-hoc comparisons were performed using Tukey’s test to determine significant differences between group means. A significant threshold of *p* < 0.05 was applied. All statistical analyses were conducted using GraphPad Prism (version 10.0.0, GraphPad Software, Boston, Massachusetts, USA). Each experimental treatment was conducted in biological triplicate, with two technical replicates per biological replicate (*n* = 6). Results are presented as mean values ± standard deviation (SD).

## Results and discussion

### Evaluation of bacterial viability following CAP treatment

**Fig. 1:**
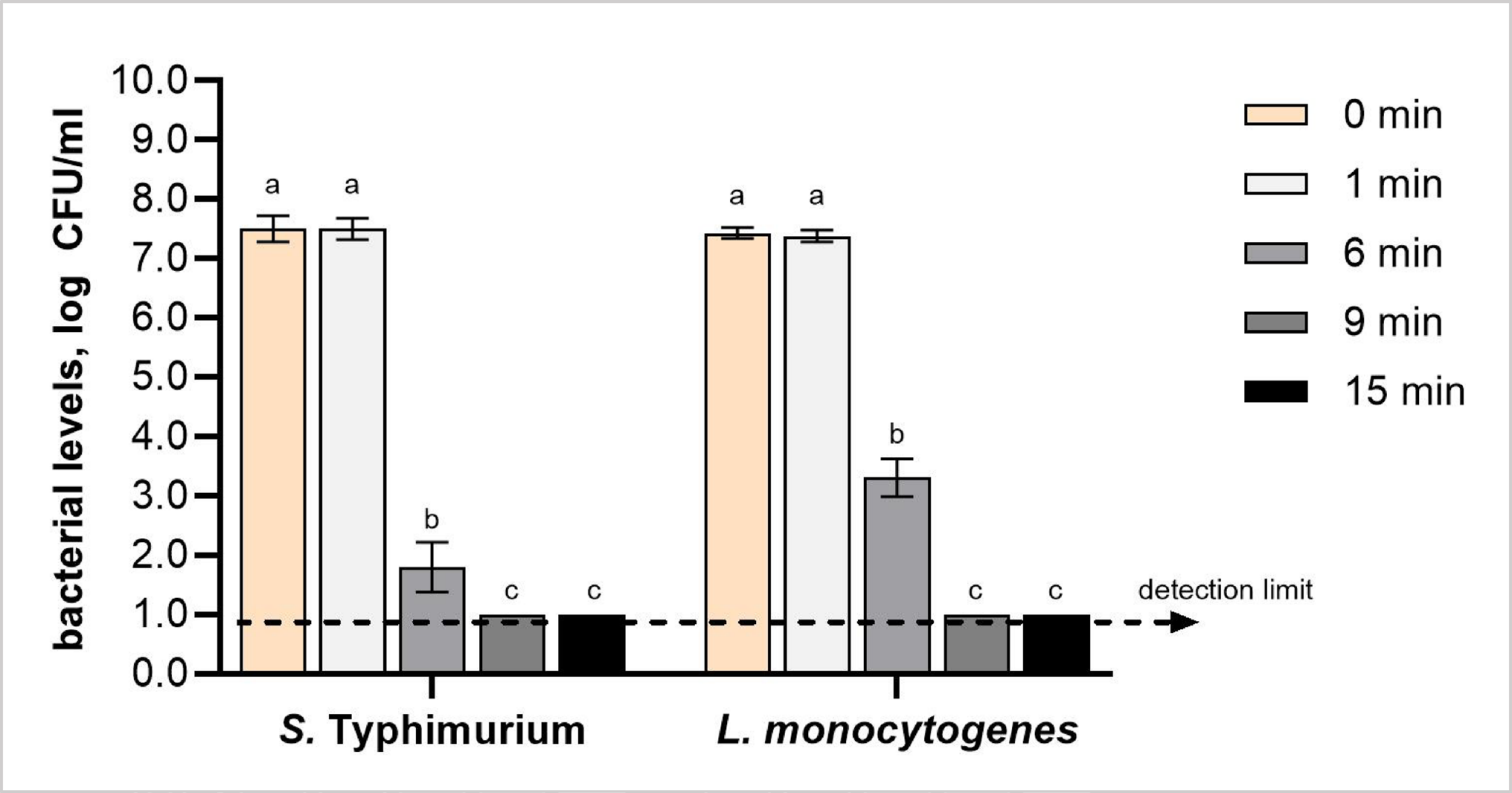
Viable counts of *Salmonella* Typhimurium and *Listeria monocytogenes* following cold atmospheric plasma (CAP) treatment at 0 (untreated), 1, 6, 9 and 15 min. Bacterial levels are expressed as log CFU/mL. The dashed line represents the detection limit of the plating method (> 1 log CFU/mL). Different letters above the bars (a–c) indicate statistically significant differences between treatment times for each bacterial species (*p* < 0.05). Error bars represent standard deviations. Each treatment was conducted in triplicate and independently replicated twice (*n* = 6)

The impact of CAP treatment on the viability of *S.* Typhimurium and *L. monocytogenes* was assessed during a range of treatment times (0–15 min) using selective agar plating (Fig. 1). At 0 min of CAP treatment, *S.* Typhimurium and *L. monocytogenes* had similar initial levels of approximately 7.5 log CFU/mL.

Following 1 min of CAP treatment, there was no reduction in viable counts for either pathogen (*p* > 0.05). A significant reduction in viable counts of *S.* Typhimurium and *L. monocytogenes* was found after 6 min treatment, resulting in reductions of 5.7 log CFU/mL and 4.1 log CFU/mL, respectively, compared to 0 min samples. For 9 min treatment, viable counts of both pathogens remained below the detection limit of the selective agar plating method (1 log CFU/mL), indicating inactivation ≤ 6.4 log CFU/mL. A significantly higher reduction in bacterial counts with increasing treatment durations (0 min, 1 min < 6 min < 9 min, 15 min) highlights the cumulative and time-dependent antimicrobial effectiveness of CAP, which can be attributed to the progressive accumulation of reactive species which induce greater structural damage and result in enhanced microbial inactivation (Harikrishna et al. 2023).

The PDD CAP generator produces plasma enriched in ozone, nitrogen dioxide, and nitric oxide which, upon contact with bacterial cells, oxidatively cleave structural bonds in membrane constituents (e.g., C–O, C–C), thereby compromising bacterial viability (González-González et al. 2021; Timmerman et al. 2021; Korzec et al. 2020a, b). Moreover, variations in the oxidative reactivity and lifespans of reactive species contribute to time-dependent changes in the chemical composition of CAP, thereby supporting the link between treatment duration and its oxidative environment (Arjunan et al. 2015). For example, hydroxyl radicals are classified as short-lived species and peroxynitrite radicals as long-lived ones; their ability to modulate each other’s reactivity during CAP treatment highlights the complexity of CAP as a reactive system (Mai-Prochnow et al. 2021).

A comparable time-dependent inactivation was observed with DBD-generated CAP using helium as the working gas, which significantly reduced *Pseudomonas aeruginosa* counts by approximately 1.0, 1.3, 1.9, and 2.0 log CFU/mL after 4, 6, 8, and 10 min treatments, respectively (Shekari et al. 2025). In contrast to our findings, a study utilising micro-discharge plasma observed no significant reduction in *S.* Typhimurium and *L. monocytogenes* counts between 10 and 20 min of treatment, suggesting a time-dependent plateau in antimicrobial effectiveness (Lis et al. 2018). These results highlight how different CAP device configurations and treatment conditions affect microbial inactivation kinetics.

Considering bacterial susceptibility based on species, *S.* Typhimurium exhibited greater susceptibility to PDD-generated CAP treatment compared to *L. monocytogenes*, showing approximately 1 log CFU/g greater reduction (Fig. 1). Our observations align closely with previous findings where *S.* Typhimurium exhibited greater susceptibility than *L. monocytogenes* to DBD-generated CAP, with approximately 1 log CFU/g greater reduction after 10 min treatment (5.3 log CFU/g vs. 4.2 log CFU/g) (Lis et al. 2018). Such differences can be attributed to the architecture of bacterial cell envelopes associated with Gram type. Specifically, the thinner cell envelope of Gram-negative *S.* Typhimurium facilitates easier penetration and greater susceptibility to oxidative damage induced by CAP-generated reactive species (Mai-Prochnow et al. 2016). Conversely, the thicker peptidoglycan layer present in the Gram-positive *L. monocytogenes* may provide enhanced structural resilience, preventing membrane disruption and intracellular stress (Jordan et al. 2008; Mai-Prochnow et al. 2016).

Although differences in Gram type were found to influence bacterial susceptibility to CAP (Jordan et al. 2008; Mai-Prochnow et al. 2016), the application of DBD-generated CAP using ambient air reduced *Staphylococcus aureus* (Gram-positive) and *Escherichia coli* (Gram-negative) to the same levels by over 10.0 log CFU/mL following 10 min treatment (Burts et al. 2009). These results indicate that extended CAP exposure and the use of ambient air may neutralise structural differences linked to Gram type, leading to comparable antibacterial effectiveness.

### Oxidative stress and lipid peroxidation assays

To investigate the role of oxidative damage in bacterial inactivation following CAP treatment, intracellular ROS accumulation and lipid peroxidation markers were quantified. ROS are one of the main active agents through which CAP exerts its antibacterial effects, due to their high reactivity and potential to cause oxidative damage to lipids, proteins, and nucleic acids in planktonic bacteria and biofilms (Schieber and Chandel 2014) is present in the text, but reference is missing in the reference section. Could you please provide the reference or delete the text citation." id="308232_307232">AQ2018). MDA is produced using lipid oxidation, and it subsequently diffuses into the intracellular environment and contributes to continued cellular damage (Alkawareek et al. 2014). The peroxide value reflects the early formation of oxidative products and provides insight into the cell oxidative stress response capacity. These markers provide evidence of cellular damage that contributes to impaired viability and ultimately leads to cell death (Van Acker and Coenye 2017).

#### Evaluation of oxidative stress assessed by intracellular ROS accumulation

**Fig. 2:**
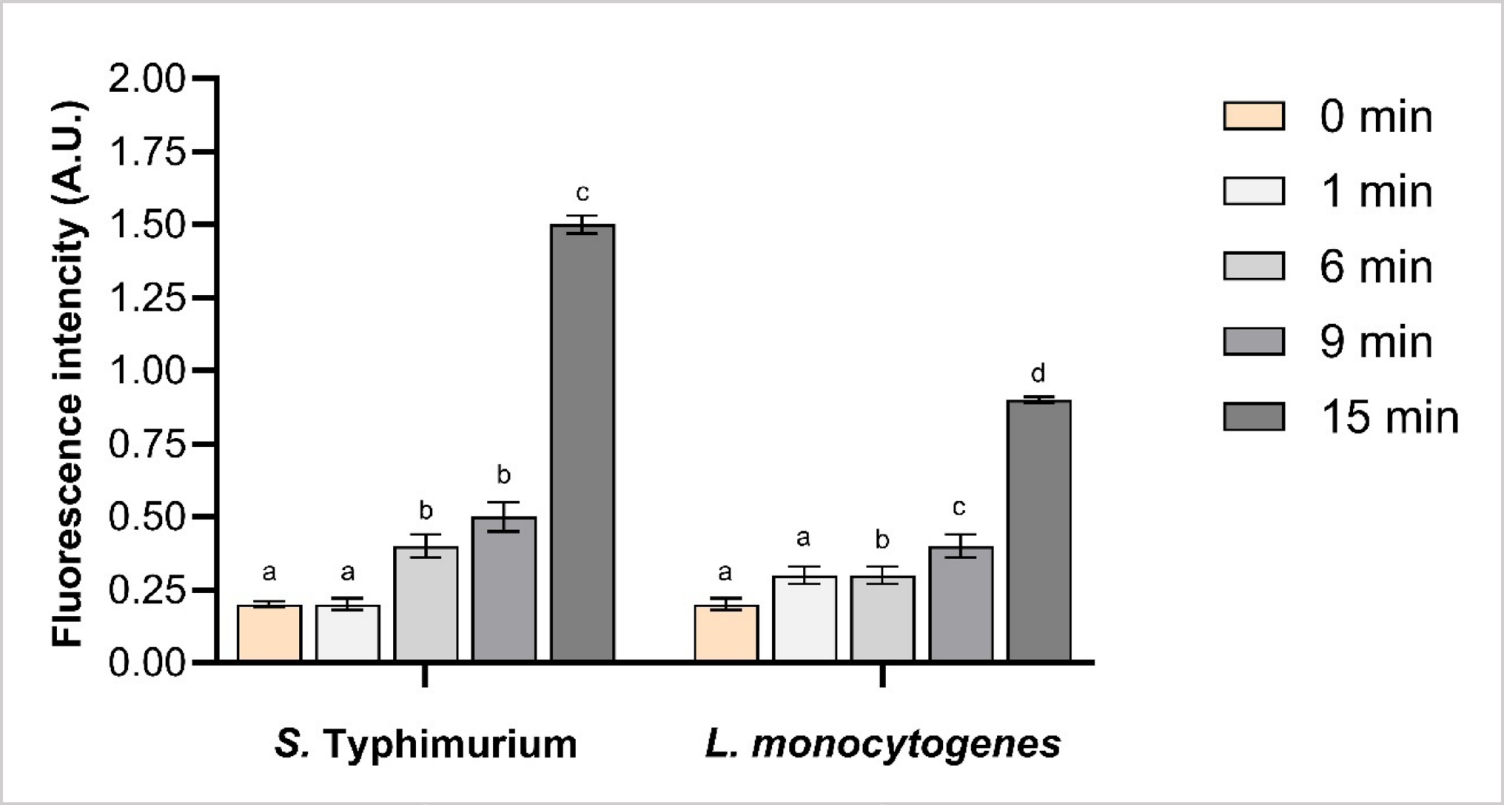
Intracellular reactive oxygen species (ROS) levels in *Salmonella* Typhimurium and *Listeria monocytogenes* following cold atmospheric plasma (CAP) treatment for 0 (untreated), 1, 6, 9 and 15 min. Different letters above the bars (a–d) indicate statistically significant differences between treatment groups (*p* < 0.05). Error bars represent standard deviations. Each treatment was conducted in triplicate and independently replicated twice (*n* = 6)

The impact of CAP treatment on intracellular oxidative stress was evaluated by quantifying intracellular ROS accumulation in *S.* Typhimurium and *L. monocytogenes* (Fig. 2). At 0 min treatment, both species exhibited low ROS levels, with optical density (OD) values of approximately 0.22 nm, respectively. No significant increase in ROS accumulation was observed after 1 min of CAP treatment in either species (*p* > 0.05).

In *S.* Typhimurium, intracellular ROS levels significantly increased by 132% at 6 min treatment, by 155% at 9 min, and peaked at 591% at 15 min (*p* < 0.05) compared to 0 min treatment. A similar time-dependent increase was observed in *L. monocytogenes*, with ROS levels rising by 73% at 6 min, 123% at 9 min, and reaching 314% at 15 min (*p* < 0.05). Considering bacterial susceptibility based on species, there was significantly higher ROS accumulation in *S.* Typhimurium compared to *L. monocytogenes* at the 15 min treatment point, indicating a greater susceptibility of the former to CAP-induced oxidative stress (Fig. 2).

Previous studies have demonstrated that various strains of *L. monocytogenes* exhibit significant increases in intracellular ROS levels following 1, 3, and 5 min of DBD treatment, with a clear time-dependent response, reaching approximately 400%, 420%, and 700% increases, respectively (Patange et al. 2019). Similarly, *Staphylococcus aureus* demonstrated significantly elevated intracellular ROS levels after 1, 2 and 2.5 min DBD-generated CAP treatment, indicating a cellular oxidative response to plasma treatment (Lunder et al. 2024). Pulsed corona discharge treatment for 30 s led to a 255% increase in intracellular ROS in *Pseudomonas aeruginosa* biofilms, highlighting the rapid oxidative response induced by CAP (Lavrikova et al. 2025).

Overall, bacteria exhibit an inherent resistance to reactive species through both enzymatic and nonenzymatic antioxidant defence mechanisms (Shimizu and Matsuoka 2019). However, our findings demonstrate a significant increase in ROS levels, indicative of a compromised antioxidant system under CAP-induced oxidative stress. Moreover, increased ROS accumulation in Gram-negative *Salmonella* Typhimurium under prolonged CAP treatment, compared to Gram-positive *Listeria monocytogenes*, may indicate structural and functional differences in their antioxidant defence systems. Previous findings indicate that CAP-induced oxidative stress, which led to ROS accumulation, can vary depending on bacterial species, CAP device type, and bacterial physiological state (e.g., planktonic vs. biofilm) (Lavrikova et al. 2025; Lunder et al. 2024; Patange et al. 2019).

#### Assessment of lipid peroxidation through MDA quantification

**Fig. 3:**
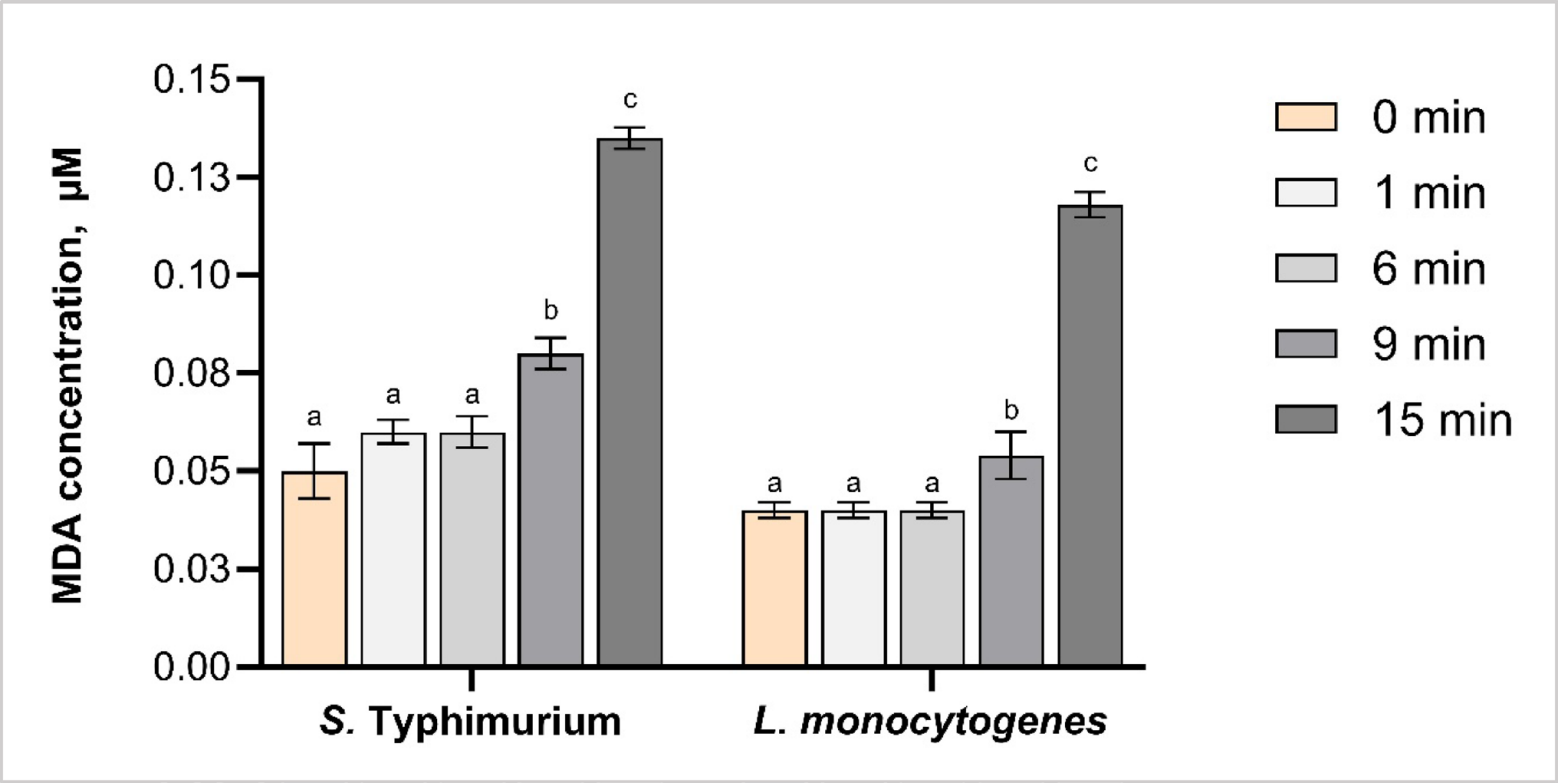
Malondialdehyde (MDA) levels in *Salmonella* Typhimurium and *Listeria monocytogenes* following cold atmospheric plasma (CAP) treatment for 0 (untreated), 1, 6, 9 and 15 min. MDA concentration was used as an indicator of lipid peroxidation. Different letters above the bars (a–c) indicate statistically significant differences between treatment times within each bacterial species (*p* < 0.05). Error bars represent standard deviations. Each treatment was conducted in triplicate and independently replicated twice (*n* = 6)

The extent of lipid peroxidation induced by CAP treatment was assessed through MDA levels in *S.* Typhimurium and *L. monocytogenes* (Fig. 3). At 0 min treatment, both species exhibited low MDA concentrations (~ 0.05 µL). No significant changes were observed following 1 and 6 min treatments in either species (*p* > 0.05). In contrast, previous studies reported a 300% increase in MDA levels in *S.* Typhimurium following 5 min of DBD-generated CAP treatment using air as the working gas, suggesting that different CAP application conditions influence the intensity of lipid oxidation (Lv and Cheng 2022).

A significant increase in MDA levels was detected following 9 and 15 min treatments, with *S.* Typhimurium showing increases of ~ 40% and 200% and *L. monocytogenes* showing ~ 30% and 170%, respectively (Fig. 3). This time-dependent effect can be attributed to the differing redox potentials and lifespans of reactive species, which collectively influence the composition and functional properties of CAP (Kondeti et al. 2018). Among ROS and RNS, hydroxyl radicals (•OH) and peroxynitrite (ONOO⁻) have the highest redox potential and exhibit strong oxidative capacity; however, •OH has a relatively short lifespan, whereas ONOO⁻ demonstrates greater stability and a comparatively longer lifespan (Arjunan et al. 2015; Mai-Prochnow et al. 2016; Zhang et al. 2023). Consequently, reducing CAP exposure time may limit the formation of short-lived but highly reactive species such as •OH, potentially diminishing the overall antibacterial effectiveness of CAP treatment.

Consistent reports of time-dependent lipid peroxidation in *Escherichia coli* following DBD-generated CAP treatment, highlights oxidative membrane damage as a central mechanism of CAP antibacterial action. Significant increases in MDA levels were observed after 12, 24, and 60 s treatments, with up to 200% increase at 60 s, indicating rapid induction of lipid oxidation (Joshi et al. 2011). Consistent with reported findings, DBD-generated CAP treatments lasting between 30 and 150 s significantly elevated MDA levels in *E. coli* following, highlighting the role of CAP exposure time in cell response (Alkawareek et al. 2014). These results overall confirm that membrane lipids are susceptible to oxidative stress induced by prolonged CAP exposure, leading to lipid peroxidation and the formation of MDA as an oxidation product. Moreover, MDA exhibits oxidative reactivity towards intracellular proteins and DNA, thereby extending CAP-induced cellular damage beyond membrane-level disruption (Kondeti et al. 2018).

Species-specific differences in MDA accumulation were found only after 15 min of treatment, with *S.* Typhimurium exhibiting approximately 30% higher levels than *L. monocytogenes*. This observation aligns with prior findings showing greater lipid peroxidation in the Gram-negative *Escherichia coli* compared to the Gram-positive *L. monocytogenes* following 5 min of DBD treatment (Olatunde et al. 2019). The higher susceptibility of Gram-negative bacteria to CAP-induced lipid oxidation has been attributed to their outer membrane, which contains lipopolysaccharides that are more prone to oxidative exposure than the thick peptidoglycan layer found in Gram-positive species (Zhao et al. 2017).

Overall, current findings highlight lipid peroxidation as a key mechanism contributing to the antimicrobial effectiveness of CAP-PDD treatment. The higher levels of MDA at prolonged treatment indicate a progressive oxidative effect, resulting from continuous ROS and RNS generation and cumulative membrane damage. When integrated with previous findings of decreased viability and increased membrane permeability, the results highlight membrane-targeted oxidative damage as a key contributor to CAP-induced bacterial inactivation.

#### Assessment of lipid peroxidation through peroxide value measurement

**Fig. 4:**
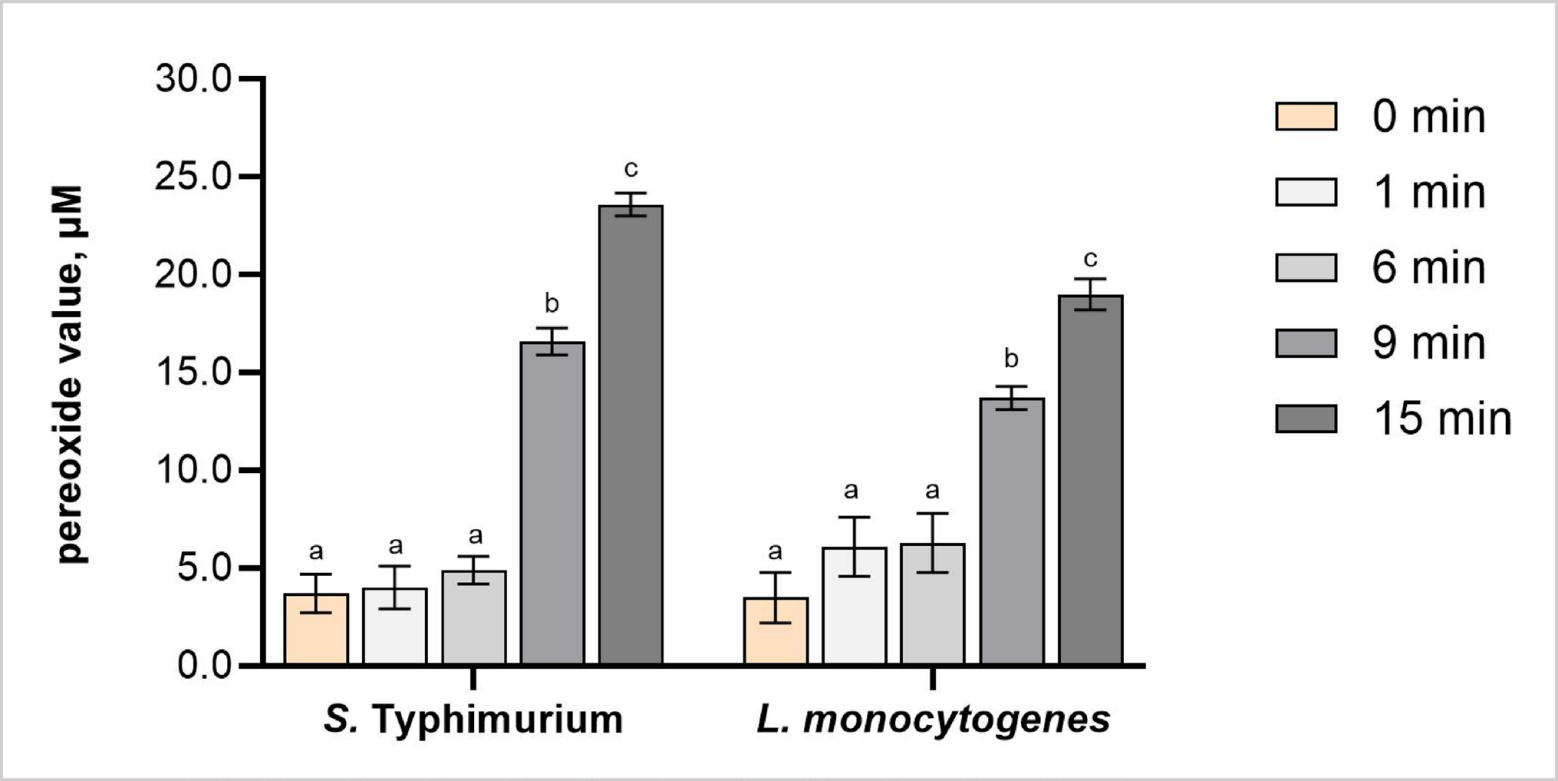
Peroxide value (PV) levels in *Salmonella* Typhimurium and *Listeria monocytogenes* following cold atmospheric plasma (CAP) treatment for 0 (untreated), 1, 6, 9 and 15 min. Different letters above the bars (a–c) indicate statistically significant differences between treatment times within each bacterial species (*p* < 0.05). Error bars represent standard deviations. Each treatment was conducted in triplicate and independently replicated twice (*n* = 6)

Peroxide value assessment further demonstrated time-dependent oxidative stress in *S.* Typhimurium and *L. monocytogenes* following PDD-generated CAP treatment. At 0 min treatment, both species exhibited low PVs, with *S.* Typhimurium and *L. monocytogenes* showing initial concentrations of approximately 5.6 µM and 4.4 µM, respectively. No significant changes were observed after 1–6 min of treatment for either species (Fig. 4). However, peroxide accumulation significantly increased from 9 min in *S.* Typhimurium (by 190%) and *L. monocytogenes* (by 260%), peaking at 373% and 410% µM, respectively, after 15 min of CAP treatment.

Species-specific differences in PV accumulation were not significant at 1, 6, and 9 min, suggesting comparable oxidative stress responses between *S.* Typhimurium and *L. monocytogenes* during early stages of CAP exposure. However, at 15 min, *S.* Typhimurium showed approximately 40% higher PV levels (Fig. 4), indicating a shift in oxidative damage once bacterial viability had been reduced. These findings suggest that *S.* Typhimurium is more susceptible to CAP-induced oxidative stress, likely due to structural differences between Gram-negative and Gram-positive bacterial cell envelopes (Mai-Prochnow et al. 2016). Particularly, the outer membrane of Gram-negative bacteria contains unsaturated lipids and lipopolysaccharides, which are sensitive to peroxidation, whereas the thick peptidoglycan layer of Gram-positive bacteria mitigates oxidative damage by limiting the diffusion of reactive species (Han et al. 2016; Mai-Prochnow et al. 2016).

Peroxide is a long-lived reactive oxygen species with a high redox potential, which contributes to the antimicrobial effectiveness of CAP (Kondeti et al. 2018). Additionally, hydrogen peroxide can absorb UV energy produced during CAP treatment, leading to its decomposition into highly reactive hydroxyl radicals that further enhance the antibacterial effect (Nicol et al. 2020). Therefore, time-dependent increases of approximately 180%, 218%, and 305% in intracellular peroxide levels in *Pseudomonas fluorescens* and *Pseudomonas putida* following 1, 2, and 3 min of DBD treatment can be attributed not only to prolonged exposure to ROS but also to UV radiation, which enhances the oxidative reactivity of ROS (Hu et al. 2025).

### Analysis of CAP-induced alterations in metabolic activity, membrane integrity, and cell morphology

To further elucidate the mechanisms underlying CAP-induced bacterial inactivation, assessments of metabolic function, membrane integrity, and cell morphology were conducted. CAP-generated reactive species can disrupt essential metabolic pathways, compromise cell membranes, and induce structural damage, ultimately leading to cell death (Mai-Prochnow et al. 2016). Together, these assays offer a comprehensive understanding of the CAP multi-target mechanism at both biochemical and structural levels.

#### CAP-induced changes in metabolic activity measured by MDH assay

To assess the impact of CAP treatment on bacterial metabolic function, malate dehydrogenase activity was measured as a marker of enzymatic activity and cellular respiration (Wolyniak et al. 2024). MDH functions as a central enzyme in the tricarboxylic acid (TCA) cycle and plays a crucial role in cellular energy metabolism (Wolyniak et al. 2024). A reduction in MDH activity reflects CAP-induced metabolic impairment, offering additional insight into sublethal cellular damage.

**Fig. 5:**
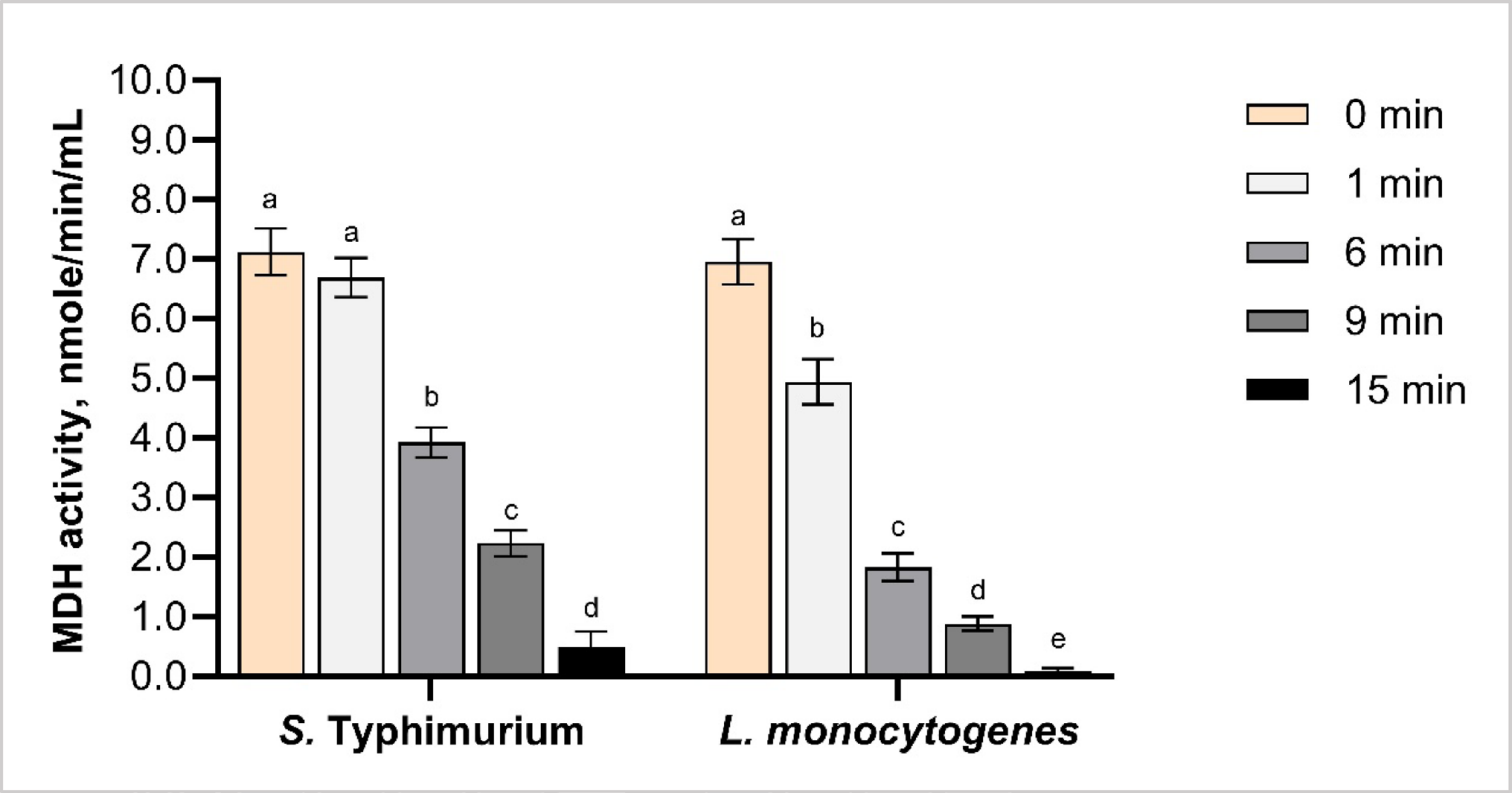
Malate dehydrogenase (MDH) activity in *Salmonella* Typhimurium and *Listeria monocytogenes* following cold atmospheric plasma (CAP) treatment for 0 (untreated), 1, 6, 9 and 15 min. Different letters above the bars (a–d) indicate statistically significant differences between treatment times within each bacterial species (*p* < 0.05). Error bars represent standard deviations. Each treatment was conducted in triplicate and independently replicated twice (*n* = 6)

CAP treatment for 1 min had no significant effect on MDH activity in *S.* Typhimurium and *L. monocytogenes*, indicating that bacterial metabolic function remained intact (Fig. 5). A statistically significant reduction in MDH activity was observed from 6 min treatment, with a further significant reduction at 9 min, and activity in both species decreasing below 1.0 nmol/min/mL following 15 min exposure. The reduction of enzyme activity observed following over 6 min treatment suggests that oxidative stress and membrane disruption, evident from ROS (Fig. 2), MDA (Fig. 3), and PV (Fig. 4) results, may collectively impair enzymatic function either by direct modification of active enzyme sites or by compromising cellular structures essential for enzyme stability. Given MDH central role in the TCA cycle and integration with other metabolic pathways, inhibition of the enzyme activity would significantly compromise adenosine triphosphate (ATP) synthesis and redox homeostasis, ultimately contributing to metabolic failure and cell death (Takahashi-Íñiguez et al. 2016; Wolyniak et al. 2024).

Moreover, exposure to ROS and RNS can shift the cellular microenvironment towards acidity, consequently reducing MDH activity by altering the optimal pH required for its function (Olszewski et al. 2014; Zaretckii et al. 2024). Particularly, the MDH enzyme in *Thermoleophilum album* has maximum activity at pH 7.5 and 60 ° C, with stability across a wide temperature range (30–95 ° C), though its catalytic activity is limited to a narrow pH range. (Novotny and Perry 1990). This suggests that time-dependent decline in MDH activity can be linked to prolonged CAP exposure, which results in cumulative acidification of the cell microenvironment.

Considering species-specific effects, MDH activity reduced to a greater extent in *L. monocytogenes* than in *S.* Typhimurium, with values falling below 2 nmol/min/mL from 9 min of CAP exposure. In contrast, *S.* Typhimurium showed comparable enzymatic inactivation only after 15 min, suggesting species-specific changes in metabolic susceptibility to CAP treatment. Similar reductions in MDH activity were reported across various bacterial species following CAP treatment. Consistent time-dependent decreases in MDH activity were found in *Pseudomonas fluorescens* and *Pseudomonas putida*, with reductions of approximately 40%, 60%, and 80% following 1, 2, and 3 min of nitrogen-based DBD treatment, respectively, confirming the accumulative impact of CAP on bacterial metabolic function (Hu et al. 2025). Further, *S. enteritidis* and *L. monocytogenes* were treated using DBD technology with atmospheric air, demonstrating both time- and frequency-dependent reductions in MDH activity. Particularly, *S. enteritidis* showed baseline activity of ~ 15 U/mg protein, with decreases ranging from ~ 6 to 14.5 U/mg depending on treatment duration (1–3 min) and voltage (60–80 kV) (Fu et al. 2022). *L. monocytogenes*, which exhibited lower initial activity, also showed progressive enzyme inhibition across all voltage levels studied (Fu et al. 2022).

#### CAP-induced membrane damage assessed by propidium iodide staining

To further characterise the effects of CAP treatment on bacterial viability, membrane integrity was evaluated using propidium iodide (PI) staining. PI selectively penetrates cells with compromised membranes, indicating membrane damage and associated viability loss (Crowley et al. 2016). When combined with culture-based enumeration and oxidative stress markers, it provides complementary insights into CAP-induced cellular damage. PI selectively enters cells with damaged membranes and binds to cell DNA, serving as a reliable indicator of membrane integrity and associated loss of viability (Crowley et al. 2016).

**Fig. 6:**
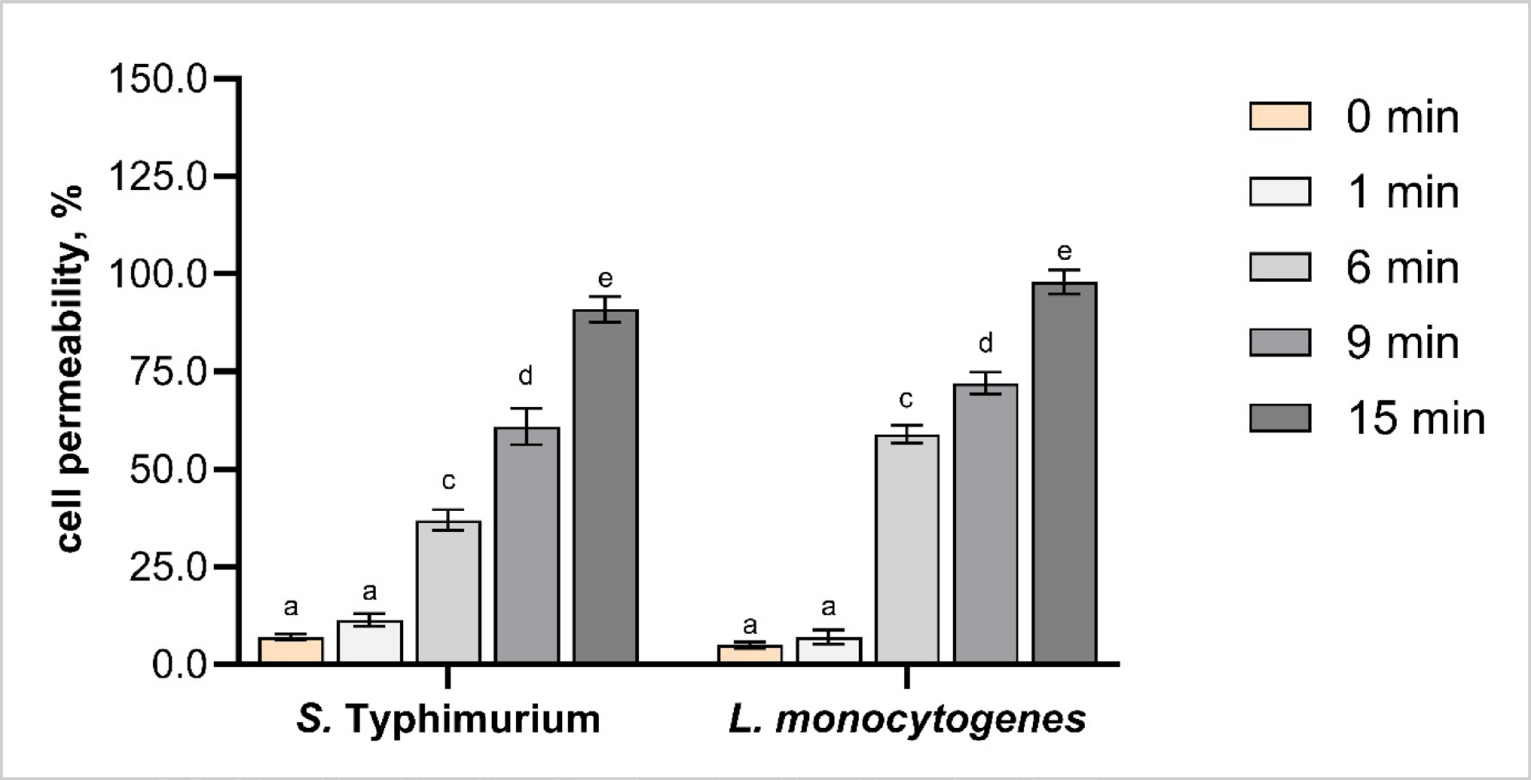
Propidium iodide (PI) uptake in *Salmonella* Typhimurium and *Listeria monocytogenes* cells following cold atmospheric plasma (CAP) treatment for 0 (untreated), 1, 6, 9 and 15 min. PI permeation was standardised against 70% propanol-treated cells, which were set as 100% PI uptake. Different letters above the bars (a–e) indicate statistically significant differences between treatment times within each bacterial species (*p* < 0.05). Error bars represent standard deviations. Each treatment was conducted in triplicate and independently replicated twice (*n* = 6)

Propidium iodide staining revealed a time-dependent increase in membrane permeability in *S.* Typhimurium and *L. monocytogenes* following PDD-generated CAP treatment. Minimal PI uptake was observed at 0 min treatment (7% for *S.* Typhimurium, 5% for *L. monocytogenes*), consistent with intact membranes in untreated cells. No significant changes in membrane permeability were found after 1 min treatment (Fig. 6). However, prolonged treatment significantly increased PI uptake in both species, with maximal values (96–100%) reached after 9–15 min. These findings confirm that CAP compromises membrane integrity in a time-dependent manner, contributing directly to bacterial inactivation.

A similar time-dependent increase in membrane permeability was observed in *L. monocytogenes* following in-package dielectric barrier discharge (DBD) plasma treatment of ham, with more pronounced damage at longer exposure times (1–10 min) (Yadav and Roopesh 2023).

A similar time-dependent increase in membrane-permeability was observed in *L. monocytogenes* following in-package DBD plasma treatment of ham (1–10 min), with greater damage reported at longer treatment durations (Yadav and Roopesh 2023). CAP treatment was found to disrupt multiple intracellular processes in *L. monocytogenes*, including oxidative stress responses, DNA repair mechanisms, and central metabolic pathways (Pan, Cheng and Sun 2022). Their multi-omics analysis revealed perturbations in methionine metabolism, glutathione metabolism, and the pentose phosphate pathway, all of which contribute to redox imbalance and reduced cell viability (Pan et al., 2022). Although our study did not include transcriptomic data, the observed increase in PI uptake, together with previously reported ROS, MDA, and PV accumulation, supports that membrane damage is both a primary and downstream effect of CAP-induced oxidative stress.

Regarding species-specific differences, *L. monocytogenes* exhibited significantly higher PI permeability than *S.* Typhimurium at 6 and 9 min of CAP treatment (Fig. 6), whereas no significant differences were observed at 1 and 15 min, indicating comparable levels of membrane damage at the initial and prolonged exposure times. According to a different study, there were DNA lesions in *L. monocytogenes* following 0–120 s DBD treatment, which highlights the role of CAP in membrane compromise, suggesting that membrane permeability enables ROS penetration and subsequent intracellular damage (Pan et al., 2022). Moreover, a 10 min DBD treatment showed an induction of DNA leakage and structural degradation in *L. monocytogenes* and S. Typhimurium biofilms, offering direct evidence of DNA instability and disruption of cellular homeostasis (Pan et al., 2022). The relationship between CAP treatment time and PI uptake supports the hypothesis that loss of membrane integrity is a critical step in the bactericidal mechanism of CAP treatment.

#### CAP-induced morphological damage revealed by SEM imaging

**Fig. 7:**
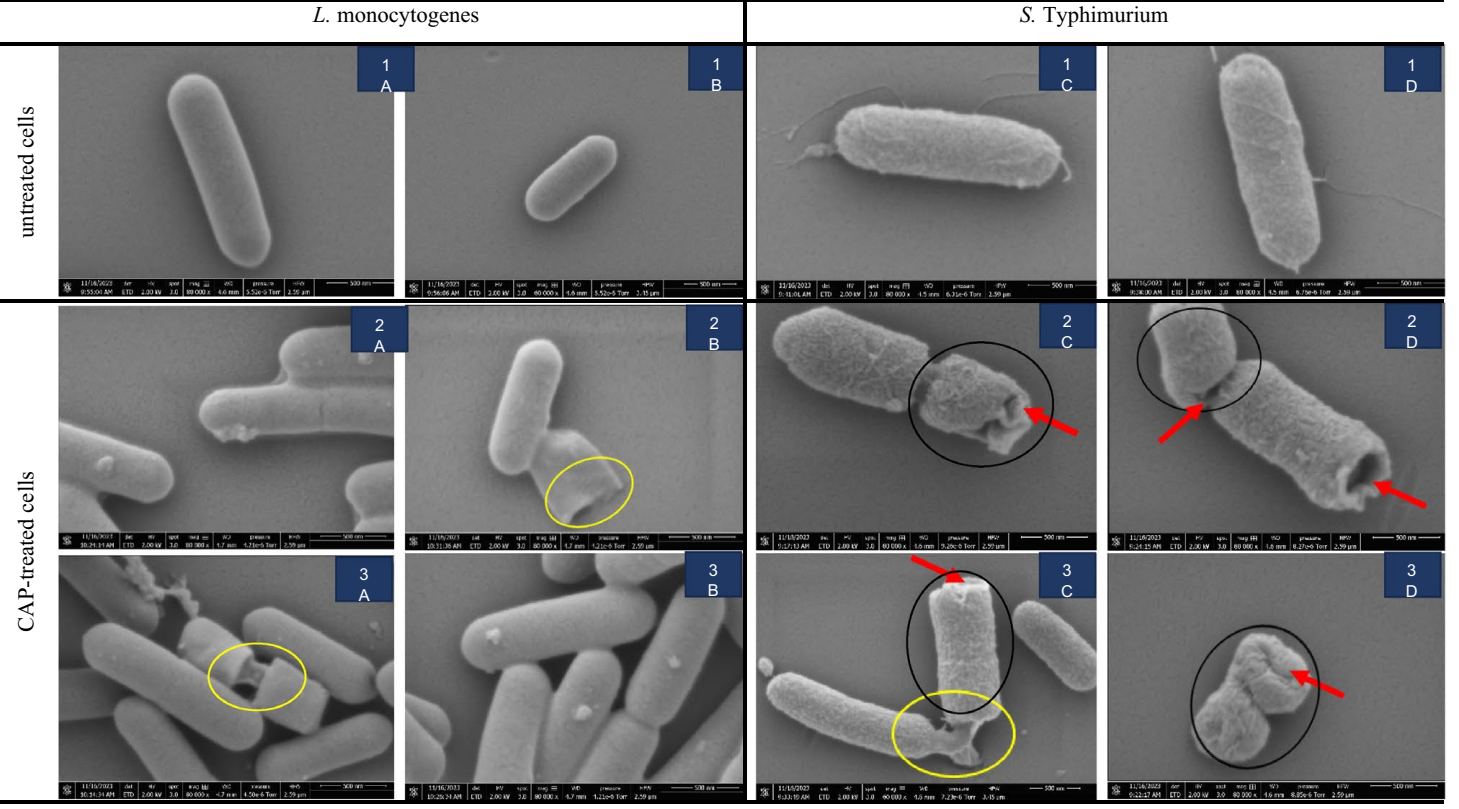
SEM micrograph of *Listeria* monocytogenes (A, B) and *Salmonella* Typhimurium (C, D) planktonic cells untreated and treated with cold atmospheric plasma (CAP-treated for 6 min). Red arrows indicate pores or perforations on the surface, yellow outlining indicates structural integrity damage leading to cytoplasm leakage, and black outlining indicates loss of regular shape. The scale bar represented 500 nm.

To assess structural damage induced by CAP treatment, scanning electron microscopy (SEM) was used to evaluate cell morphology and integrity. SEM imaging revealed distinct morphological alterations in *S.* Typhimurium and *L. monocytogenes* following 6 min PDD-generated CAP treatment (Fig. 7).

Untreated cells exhibited intact, rod-shaped morphologies with smooth and uniform surfaces, consistent with well-preserved cellular structure (Figs. 7.1a–d). In contrast, CAP-treated cells showed surface deformities, including porosity and shrinkage (Figs. 7.2a–d and 7.3a–d). Structural alterations were more diverse in *S.* Typhimurium, which showed extensive surface disruption, cytoplasmic leakage, fragmentation, and cellular shrinkage (Figs. 7.2c–d and 7.3c–d). In contrast, *L. monocytogenes* displayed a narrower range of morphological changes (Figs. 7.2a–b and 7.3a–b), which was primarily cytoplasmic leakage.

Similar CAP-induced damage was in *E. coli*, including membrane disruption and cytoplasmic leakage after 10 min DBD-generated CAP treatment (Laroussi et al. 2003). Similarly, plasma jet application for 1 min caused significant structural changes such as cell wall collapse, ruptures, and deformation in Gram-negative *E. coli* and *Pseudomonas aeruginosa*. Particularly, *E. coli* showed greater cell damage, indicating higher susceptibility to CAP compared to *P. aeruginosa* (Dahle et al. 2024). This distinction between Gram types was also reported in a study assessing *Salmonella* Typhi, *Vibrio cholerae*, *Bacillus cereus*, and *L. monocytogenes* after 2 min DBD treatment. SEM analysis revealed species-specific morphological disruption, with *S.* Typhi showing the highest degree of structural disruption (Khatami et al. 2023). These findings support our observation that outer membrane structure plays a critical role in determining bacterial susceptibility to CAP-induced physical damage, with Gram-negative species generally showing greater susceptibility (Figs. 1-7).

Overall, assessments of metabolic activity, membrane integrity, and morphological changes revealed distinct response mechanisms of *L. monocytogenes* and *S.* Typhimurium to PDD-generated CAP treatment. *L. monocytogenes* displayed rapid metabolic reduction and increased membrane permeability despite its thicker Gram-positive cell wall, suggesting earlier intracellular disruption. In contrast, the Gram-negative *S.* Typhimurium showed delayed metabolic impairment but more pronounced structural damage, including fragmentation and shrinkage, indicative of greater outer membrane susceptibility. These findings suggest that *L. monocytogenes* and *S. Typhimurium* indicate species-specific susceptibilities to PDD-generated CAP, with *S. Typhimurium* demonstrating greater structural vulnerability, likely reflecting fundamental differences in cell envelope architecture and stress response.

## Conclusions

This study demonstrated that CAP treatment effectively inactivated *Salmonella* Typhimurium and *Listeria monocytogenes* through a time-dependent and multi-mechanistic mode of action. CAP application significantly reduced bacterial counts, compromised membrane integrity, increased intracellular ROS, triggered lipid peroxidation, and impaired metabolic activity, as evidenced by decreased malate dehydrogenase function. Distinct responses between the two bacterial species highlighted the critical role of the structure of the cell envelope in affecting susceptibility to CAP and underscored the importance of optimising treatment parameters for each pathogen.

Overall, these findings confirm that PDD-generated CAP induces microbial inactivation using the combined effects of oxidative stress, loss of membrane integrity, and disruption of metabolic pathways. This underscores the potential of PDD CAP as an effective, non-thermal antimicrobial intervention in food safety applications.

Future research should focus on integrating CAP technology into diverse food matrices to evaluate its effects on microbial ecology, oxidative stability, and product quality. Comprehensive assessments across microbiological, biochemical, and sensory parameters will be essential for regulatory approval and successful commercial implementation of CAP in food preservation.

## Data Availability

The data that support the findings of this study are not openly available due to reasons of sensitivity and are available from the corresponding author upon reasonable request.
